# Carotid Endarterectomy in a Patient with Severe Internal Carotid Artery Stenosis with Persistent Trigeminal Artery and Ischemia of the Anterior and Posterior Circulation

**DOI:** 10.1155/2017/7193734

**Published:** 2017-12-04

**Authors:** Melanie R. F. Greenway, Hussam A. Yacoub, Shweta Varade, Yevgeniy Isayev

**Affiliations:** ^1^Morsani College of Medicine SELECT Program, Lehigh Valley Health Network and University of South Florida, Tampa, FL, USA; ^2^Lehigh Valley Physician Group-Neurology, Lehigh Valley Health Network, 1250 S. Cedar Crest Blvd., Suite 405, Allentown, PA 18103, USA

## Abstract

Occurrence of cerebral ischemia in the posterior circulation as a result of severe internal carotid artery disease and persistent trigeminal artery is rare. An 81-year-old man with medical history of hypertension and ischemic stroke presented with dizziness, nausea, and mild dysarthria. Magnetic resonance imaging of the brain revealed acute infarcts in the left internal carotid artery territory. CT angiogram revealed a persistent trigeminal artery (PTA) and severe atherosclerosis. The patient developed new neurological symptoms and repeat imaging revealed new acute infarcts in the PTA distribution. After undergoing a left carotid endarterectomy with no complications, the patient was discharged to a skilled nursing facility with no recurrence of ischemic stroke. This case adds a rare complication of an infrequent vascular anomaly to the limited body of the literature.

## 1. Introduction

Persistent trigeminal artery (PTA) is the most common and most cephalad-located persistent carotid-basilar arteries anastomosis with a reported incidence of 0.1% to 0.6% [1, 2] in the general population. A PTA is typically found incidentally on angiographic studies. The trigeminal artery appears in the 3-mm embryonic stage and supplies the precursors of the basilar artery but normally regresses as early as the 11.5-mm to 14-mm embryonic stage [3]. The trigeminal artery may persist into adult life as a branch of the cavernous portion of the internal carotid artery (ICA); however, occurrence of cerebral ischemia in the posterior circulation secondary to severe ICA disease and PTA disease is rare. We report a case of an 81-year-old man with a PTA arising from the left cavernous ICA supplying the mid-distal basilar artery, with acute infarcts in both the ICA and PTA territories.

## 2. Case

A right-handed 81-year-old man with a history of ischemic stroke, hypertension, hyperlipidemia, hypothyroidism, and chronic kidney disease presented to the Emergency Department with dizziness, nausea, dysarthria, and change in mental status. Further history revealed that he had two prior ischemic strokes, in 2003 and 2007, with residual right-sided weakness. Subsequently, he had been on aspirin/dipyridamole for stroke prevention.

On initial examination, the patient was afebrile with a blood pressure of 162/81, heart rate of 77 beats per minute, and oxygen saturation of 98% on room air. He was alert and oriented to person but disoriented to location and time. On neurological examination, he was found to have poor memory, fair concentration, mild dysarthria and nonfluent aphasia, poor knowledge, and limited judgement. Pupils were equally round and reactive to light with intact extraocular movements. Facial sensation was intact bilaterally. No facial weakness was appreciated and tongue was midline on protrusion. Motor examination was nonfocal without a pronator drift. Sensory examination was intact to light touch, proprioception, and pinprick. Reflexes were symmetrical and coordination was normal.

Laboratory studies revealed a white blood cell count of 12,600/*μ*L, platelet count of 155,000/*μ*L, sodium level of 141 mEq/L, potassium level of 4.3 mEq/L, glucose level of 123 mg/dL, and serum creatinine level of 1.50 mg/dL. An electrocardiogram showed a normal sinus rhythm and a 2D echocardiogram did not reveal any valvular or wall motion abnormalities. Telemetry monitoring in the hospital did not reveal any atrial fibrillation.

**Figure 1:**
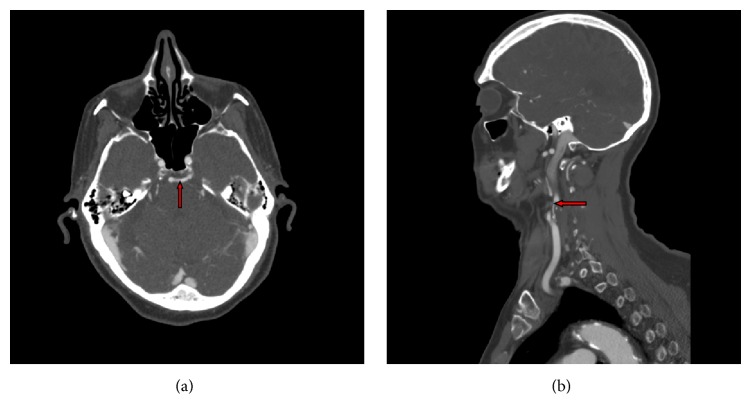
(a) A CT angiogram showing a persistent trigeminal artery arising from the left cavernous internal carotid artery and supplying the mid-distal basilar artery (red arrow). (b) A CT angiogram of the neck revealing the severely stenosed left internal carotid artery (red arrow).

An outpatient computed tomography (CT) scan showed no acute findings. CT angiogram (CTA) of the head revealed a remote infarct in the left parietal lobe and diminished flow within the distal left middle cerebral artery branches. The CTA further showed a PTA supplying the mid and distal basilar artery (Figure 1(a)) and severe atherosclerosis and stenosis of the proximal ICA (Figure 1(b)).

Magnetic resonance imaging (MRI) of the brain showed several areas of restricted diffusion in the posterior left temporal and frontal lobes, right occipital lobe, and bilateral cerebellar hemispheres consistent with subacute infarcts.

**Figure 2:**
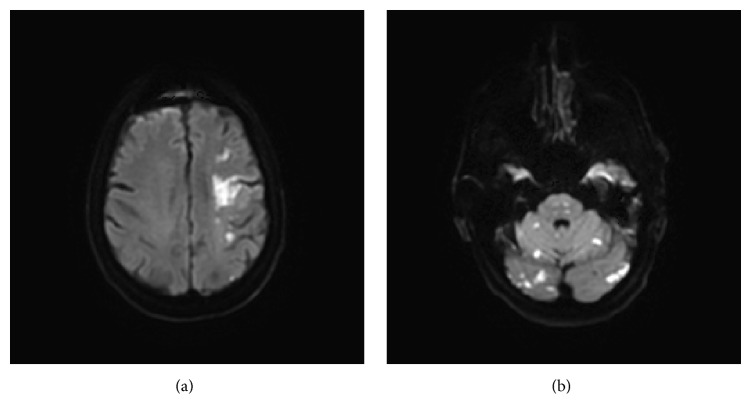
(a) An MRI of the brain revealing areas of restricted diffusion in the left carotid artery distribution. (b) MRI of the brain showing new infarcts in the superior cerebellum and occipital lobe, within the PTA territory.

Two days after admission, the patient was noted to be nonverbal and somnolent. Motor examination revealed right-sided hemiplegia. A repeat MRI of the brain showed new areas of restricted diffusion in the left frontoparietal region, and also in the brain stem, bilateral superior cerebellar, and occipital regions. Findings were most consistent with emboli originating from the severely atherosclerotic left ICA, reaching the posterior fossa via the PTA (Figure 2). After reviewing the images with a neuroradiologist, it was determined that the new acute infarcts found in the occipital lobes, superior cerebellum, and thalamus were within the PTA territory.

The patient was transferred to the neuroscience intensive care unit for monitoring with a systolic blood pressure goal of 180 to 200. A carotid duplex showed 80% to 99% stenosis of the proximal left ICA. Subsequently, the patient underwent a left carotid endarterectomy with bovine patch angioplasty one week after admission with no complications. His neurological examination improved slowly and the patient was eventually discharged to a skilled nursing facility for rehabilitation with no recurrence of ischemic stroke.

## 3. Discussion

Occurrence of cerebral ischemia in the posterior circulation as a result of severe ICA disease and PTA is rare. Ulcerative atherosclerotic disease of the internal carotid artery can be implicated as a potential source of emboli that would reach the posterior circulation through a PTA, but cases of radiographically confirmed infarcts are rarely reported.

Hwang and Kim [4] recently reported the first case of an aneurysm of the PTA that directly terminated in the cerebellar arteries and combined with multiple aneurysms. Interestingly, aneurysms of the PTA trunk are exceptionally rare and have a high risk for rupture. Other incidental findings associated with PTA include aneurysms of other cerebral vessels [5], trigeminal cavernous fistula [6], arteriovenous malformation [7], and moyamoya disease [8].

Gasecki et al. [9] reported bilateral occipital infarcts associated with carotid stenosis in a patient with a PTA. This case is an addition to the limited body of the literature that also demonstrates the utilization of carotid endarterectomy to prevent further infarcts in the anterior and posterior vascular territories in this patient population. Furthermore, this case study supports the benefit of intracranial vessel imaging in the evaluation of stroke etiology. Imahori et al. [10] reported a similar case of acute ischemic stroke involving both the anterior and posterior circulation associated with a PTA. Management involved endovascular revascularization of the acute basilar artery embolus.

We advise clinicians to carefully evaluate for anomalous connections between the ICA and the basilar arteries in any patient with posterior circulation infarcts of an unclear source and severe carotid atherosclerotic disease.
